# The Proper Time to Operate

**Published:** 1862-09

**Authors:** W. H. Atkinson


### DENTAL REGISTER OF THE WEST.

VOL. XVI.] SEPTEMBER, 1862. [No. 9.

### Original Essays and Communications.

### THE PROPER TIME TO OPERATE.

BY DR. W. H. ATKINSON.

Read before the N. Y. Society of Dental Surgeons, May 21, 1862.

THE proper time to operate is when the patient and operater are both fully ready. Just what constitutes readiness is not always apprehended by both, and sometimes not by either. Readiness on the part of the patient consists in physical and moral state, without either of which, results will be less favorable or disastrous, in the degree of unfitness of bodily or mental system.

Fitness on the part of the operator also depends upon the physical and moral constitution and state at the time.

Physical fitness of patient depends upon the degree of degeneracy that holds the dominion of the part to be operated upon and the tone of the general system or constitution, enabling it to recuperate after the shock of the operation is over.

In cases where the shock can be made very light or entirely prevented, a much lower degree of vitality may be adjudged compatible with an operation than in cases where the violence of the shock is considerable, and can not be broken in a great degree by the employment of anæsthetics.

Moral fitness of patient depends upon the confidence he has in the operator to properly execute his functions to him, in the particular case, and will be complete when all the elements of fitness are supposed to reside in the operator.

In cases where the patient has confidence only in the skill or mere ability to execute the manipulative processes, and not in the tenderness and oneness in sympathy of the operator, we shall have a much inferior degree of success displayed.

Fitness of remedies is not so readily stated so as to be fully understood; for the condition of tissue and system to which remedies are applied, and the potency of the remedy, both require to be intelligently apprehended before the compatibility or incompatibility can be ascertained.

The same remedy will be fit or unfit for use in a particular case or stage of a case, according to the concentration or attenuation of its strength or affinity to the part.

Physical fitness of operator implies a degree of health and strength commensurate with an easy and ready performance of the work without such exhaustion as to disturb the moral state.

Mental fitness implies a knowledge of the principles of anatomy, physiology and pathology of the general system and the special tissues upon which about to pass a diagnostic decision, and a detailed clearness of conception of the malady, in nature and degree of development at the time it is presented, including an understanding of all the elements of fitness and unfitness of preparation of the patient for an operation.

When all these elements of fitness are found in operator and patient, with proper instruments and remedies at hand, we are sure of good results.

But in the vast majority of cases in the practice of medicine, surgery and dentistry, we do not combine all these favorable conditions, and hence we have less than complete success in the majority of cases.

Shall we abandon our efforts to effect some good, therefore, because we can not combine all that we can conceive in each and every case? or shall we not the rather bring together as many favorable conditions as we may, and thus effect at least an approximation to the good we sought to bring about for the subject of disease?

Making close observations that we may increase our knowledge and ability for future cases, will enable us to extend the range of curability and compatibility more and more, until disease and intractable cases shall have been banished from the entire field of practice.

The readiest mode of procedure to effect this desirable end is to add moral fitness of operator and patient in each and every instance to the physical and mental compatibility before enumerated as true elements of a successful career of practice that shall finally culminate in the abnegation of the necessity for operations.

A peculiar state of recedence of vital force, heretofore called sthenic action, or an inflammation above the standard of vital action, has been particularly pointed out as the stage in which operations were peculiarly liable to eventuate unfavorably.

And hence it has been the uniform advice of competent surgeons to await the subsidence of this stage before attempting the performance of any capital operation.

Now, the advice to delay was good, but the philosophy and reasoning upon which it purports to be based is false and dangerous. As there can be no action of healthy system above the vital standard, all departure must be minor manifestations of vital function, exhausting and destructive to the whole machinery of the living economy.

That peculiarly low state of the vital powers which permits exudation and infiltrations of aqueous lymph, which is sure to precede malignant and intractable swellings and abscesses, is always an unfavorable time for surgical interference by knife or caustic or any treatment involving lesion of the debilitated parts while under undue tension, renders the circulation stagnant or irregular. Tonic and soothing applications and mechanical support are the best local resorts, with prompt nauseating internal remedies; after which evacuation of the illy or well digested matters, to free the constitution of their morbific presence, will be very advisable, if not absolutely the only way to ward off untoward or fatal results.

